# Influence of Storage Conditions on Four Chipping Potato Cultivars Developed in North Dakota

**DOI:** 10.3390/plants13202868

**Published:** 2024-10-14

**Authors:** Zhiwei Chen, Asunta L. Thompson, Jawahar Jyoti, Harlene M. Hatterman-Valenti

**Affiliations:** 1 PepsiCo Foods (China) Co., Ltd., Pingan River Front Financial Center 18F, No. 757 Meng Zi Road, Huangpu District, Shanghai 200023, China; alanchengator@gmail.com; 2 Plant Sciences Department, North Dakota State University, Fargo, ND 58108, USA; asunta.thompson@ndsu.edu (A.L.T.); jawahar.jyoti@ndsu.edu (J.J.)

**Keywords:** cold chipper, reducing sugar, chip color, Agtron value

## Abstract

Cold temperature storage (lower than 10 °C) has been used as a management strategy to extend marketability and reduce potato storage losses. However, cold temperatures may result in dark-colored chips through a process known as cold-induced sweetening (CIS). ‘Dakota Crisp’ and ‘Dakota Diamond’ are two North Dakota State University potato breeding program cultivar releases selected for cold-chipping ability with high tuber yield potential. Two-year storage trials were conducted to examine sugar development and tuber processing quality of four cultivars grown at three nitrogen rates under irrigated and non-irrigated field conditions. The two-way interaction between storage period and storage temperature was significant for sucrose content, glucose content, visual chip color, and Agtron values, indicating a difference in sugar development for each storage temperature profile. Among the four cultivars evaluated under both irrigated and non-irrigated production conditions, ‘Dakota Pearl’ accumulated significantly less sucrose and glucose compared to other cultivars under the same storage conditions. ‘Dakota Crisp’ produced acceptable chip color from 8.9 °C after long term storage, while ‘Dakota Diamond’ produced acceptable chip color from 8.9 °C for up to 6 months of storage. These results emphasize the importance of developing cultivar-specific management profiles including storage and the informational need for producers and processors in determining the best practices for individual cultivars.

## 1. Introduction

Harvested potato tubers require optimal conditions to maintain high quality during storage, because tubers are living, breathing, and biologically active organisms [1]. Storage temperature and time in storage are two important aspects of cultivar-specific management. Sugar response and potato chip appearance are affected by cultivar, as well as storage length and storage temperature [2]. The rate of conversion from starch to sugar, which affects the sugar level, is determined by ambient gasses, storage temperature, tuber maturity, growing season stresses, and physiological age [3]. Among these, storage temperature has been shown to be the most significant factor affecting the conversion rate [4]. Previous research indicates low temperatures cause increased permeability and disintegration of the amyloplast membrane, allowing enzymes to break down starch into sugars [5,6]. When stored potato tubers are processed (fried) at high temperatures, these accumulated reducing sugars react with cellular amino acids, resulting in the unfavorable non-enzymatic browning or “Maillard” reaction [7].

New potato cultivars classified as “cold-chippers” have increased market interest because they may be processed directly from colder storage temperatures (4 °C to 6 °C), with advantages including reduced disease losses, maintenance of chip quality, and extended storage period [8]. Thus, the development of cold-chipping cultivars that can be consistently processed into light-colored potato chips from colder storage temperatures (4 °C to 6 °C) is an important objective for many potato breeding programs. Today, scientists breed for and select germplasm (potential cultivar releases) considered to be cold-sweetening resistant. 

Potato germplasm has been identified with the potential for cold-sweetening resistance. Hyde and Walkof [9] reported that a potato seedling, F5208, accumulated a negligible amount of reducing sugars during cold storage. This seedling showed resistance to the starch–sugar conversion under low temperatures and was used to develop cold-sweetening-resistant chipping cultivars. Johansen [10] selected and developed a potato seedling, ND860-2, which produced acceptable color after storage at 4 °C when grown under North Dakota conditions. ND860-2 was not immune to a sugar increase in cold storage, but sugar content increases were not great enough to darken the chip color to an unacceptable level. Coffin et al. [11] reported similar results in Alberta and Manitoba, by observing acceptable chip color following 28 w storage at 6 °C. This trait, termed “cold temperature processability” (CTP), was considered to be a stable trait, since similar results were observed with ND860-2 under different environmental conditions in Ontario. Since then, ‘Snowden’, and numerous selections have been bred, which can be chip processed directly from 7.2 °C [12]. Hanneman [13,14] identified several Solanum species that could be chip processed directly from 4 °C storage. These species provide the anti-sweetening genes that are being introgressed into new processing genotypes. 

Introgression of cold-sweetening resistance genes is hampered by two major obstacles, including the fact that 70% of wild potato species are diploid [15] and they often lack tuberization under long days in northern temperate production regions [16]. These wild Solanum species may have potential crossing barriers with tetraploids, and often have lower yield potential due to late-season tuberization and bulking. Conventional potato breeding techniques are used by potato breeders to incorporate the cold-induced sweetening resistance trait into the tetraploid cultivated potato (*Solanum tuberosum* L.). Heritable variation for cold chipping has been well documented in tetraploid and diploid populations [17]. Despite this, no commercially acceptable cultivars that consistently produce acceptable chips directly from 4 °C storage have been released [16] and little research has been published on chip processing profiles. ‘Dakota Crisp’ [18] and ‘Dakota Diamond’ [19] are two cultivar releases from the North Dakota State University potato breeding program with cold chipping potential. It is important to have a better understanding of the storage sugar profiles and to develop appropriate storage management strategies for new cultivar releases. 

Nitrogen management may affect potato chip quality, including dry matter content, sugar levels and chip/processing color [20,21,22,23]. Nitrogen (N) is a phloem-mobile nutrient, which can move to tubers at harvest [24]. Consequently, N application rate and timing may affect the sugar and starch content of tubers. Nitrogen uptake profiles indicate that most N is taken up by potato plants during tuber initiation and bulking, with 60% taken up by 75 days after planting (DAP) [25]. Chemically immature tubers accumulate greater amounts of reducing sugar in storage compared to tubers properly matured and vine killed a week to 10 days before harvest [5]. Chemical maturity monitoring (CMM), the first commercially feasible method developed to measure the “chemical maturity” of potatoes in the field, is based on sugar concentrations at harvest [26]. Sowokinos and Preston [26] reported that the level of sucrose in chipping potatoes should be 1.5 mg g^−1^ or less at harvest to minimize accumulation of reducing sugars during long-term storage. Thus, the determination of sucrose content at maturity is currently used to aid plant breeders in developing potentially superior potato cultivars for processing from storage [6]. While sucrose is important, it does not directly participate in the Maillard reaction as the reducing sugars glucose and fructose do [27]; however, sucrose breaks into reducing sugars during storage and thus is a good measure for predicting chip color following storage. The objective of this research was to evaluate sugar development and chipping performance of four chipping cultivars at two locations (irrigated or non-irrigated) under varying storage temperatures and storage periods. 

## 2. Results

### 2.1. Tuber Sucrose Content

A significant four-way interaction was found for location by cultivar by storage temperature by storage period (*p* = 0.002), which resulted in three significant three-way interactions and six significant two-way interactions. The only three-way interaction that was not significant was location by cultivar by storage temperature. 

Tuber sucrose content was influenced by cultivar and the location where plants were grown (Table 1). ‘Dakota Crisp’ grown under irrigation had the highest tuber sucrose content, which was greater than other cultivar and location combinations. In contrast, ‘Dakota Diamond’ and ‘NorValley’ grown under non-irrigated conditions had the lowest tuber sucrose content. The source of the interaction was ‘Dakota Pearl’ which had a lower tuber sucrose content when plants were grown under irrigation compared to non-irrigated conditions, while the other three cultivars had increased tuber sucrose contents when plants were grown under irrigation compared to non-irrigated conditions.

Tuber sucrose content was influenced by location and tuber storage temperature (Table 2). Tubers produced under irrigation and stored at 7.2 °C had the highest tuber sucrose content, while tubers from plants grown under non-irrigated conditions and stored at 5.5 or 8.9 °C had the lowest tuber sucrose content. The source of the interaction was the lowest tuber sucrose content at 8.9 °C when plants were irrigated compared to non-irrigated conditions, while the other two storage temperatures had increased tuber sucrose content when grown under irrigated compared to non-irrigated conditions.

Tuber sucrose content was influenced by location and storage period (Table 3). Plants grown under non-irrigated conditions with tubers stored for 10 months had the highest tuber sucrose content, which was greater than any other location and storage period combination. In contrast, plants grown under non-irrigated conditions with tubers stored for 1 to 7 months had the lowest tuber sucrose contents. The source of the interaction was the 10-month storage period, which had a lower tuber sucrose content when plants were grown under irrigation compared to non-irrigated conditions; conversely, the other seven storage periods demonstrated increased tuber sucrose contents when plants were grown under irrigation compared to non-irrigated conditions.

Tuber sucrose content was influenced by cultivar and tuber storage temperature when averaged over location and years (Table 4). ‘Dakota Crisp’ tubers stored at 7.2 °C had the highest tuber sucrose content, while ‘Dakota Crisp’ and ‘Dakota Diamond’ tubers stored at 8.9 °C, and ‘NorValley’ tubers stored at 5.5 °C had the lowest tuber sucrose contents. The source of the interaction was ‘NorValley’ tubers stored at 5.5 and 8.9 °C, where tubers stored at 8.9 °C had higher sucrose content than tubers stored at 5.5 °C. In contrast, the other cultivars had tubers with higher sucrose contents when stored at 5.5 °C compared to the sucrose content from tubers of the same cultivars stored at 8.9 °C.

Tuber sucrose content was influenced by storage temperature and storage period (Table 5). Tubers stored at 7.2 °C for 10 months had the highest tuber sucrose content, while tubers stored at 8.9 °C for 3, 4, 5, or 7 months had the lowest tuber sucrose content. The source of the interaction was tuber storage at 5.5 °C; tuber sucrose content was the highest when tubers were stored for 1 month and decreased after storage for 2 and 3 months, while tubers stored at 7.2 °C had the lowest tuber sucrose content when stored for 1 month and increased after storage for 2 months and 3 months. The other source of the interaction was the slight increase in tuber sucrose content for tubers stored at 5.5 °C for 7 and 10 months, while the tuber sucrose content almost doubled for tubers stored at 8.9 °C for 7 and 10 months.

Finally, tuber sucrose content was influenced by cultivar and storage period (Table 6). ‘Dakota Pearl’ tubers stored for 10 months had the highest tuber sucrose content, higher than the tuber sucrose content for any other cultivar and storage period combination. ‘Dakota Crisp’ and ‘NorValley’ tubers stored for 10 months had the second highest tuber sucrose contents. In contrast, ‘NorValley’ tubers stored for 1, 2, 4, or 7 months and ‘Dakota Diamond’ tubers stored for 6 months had the lowest tuber sucrose content. The source of the interaction was primarily ‘NorValley’ and ‘Dakota Diamond’, where ‘NorValley’ tuber sucrose content increased 127% from 1.47 to 3.34 mg g^−1^ when tubers were stored for 7 and 10 months, respectively, while ‘Dakota Diamond’ tuber glucose content decreased 4% when tubers were stored for 7 and 10 months, respectively. The main sources of interaction were between ‘Dakota Crisp’ and ‘Dakota Pearl’ at a couple of storage periods and between ‘Dakota Diamond’ and ‘NorValley’ at a couple of storage periods.

### 2.2. Tuber Glucose Content

A significant four-way interaction was found for location by cultivar by storage temperature by storage period (*p* = 0.03), resulting in four significant three-way interactions and five significant two-way interactions. The only two-way interaction that was not significant was location by storage temperature (*p* = 0.50). 

Tuber glucose content was influenced by cultivar and the location where plants were grown (Table 1). All values exceed the desired threshold of 0.35 mg g^−1^ fresh wt. [28]. ‘Dakota Diamond’ and ‘NorValley’ plants grown under non-irrigated conditions had greater tuber glucose content than any other cultivar and location combination. In contrast, ‘Dakota Pearl’ plants grown under irrigation had the lowest tuber glucose content. The source of the interaction was ‘NorValley’, which had a greater decrease (132%) in tuber glucose content when plants were grown under irrigation compared to non-irrigated conditions, while the other cultivars had only a 43–84% decrease in tuber glucose content when plants were grown under irrigation compared to non-irrigated conditions. 

Again, tuber glucose content exceeded the desired industry threshold for the interaction of location and storage temperature. Tuber glucose content was influenced by location and storage period (Table 3). Similar to the results for sucrose, plants grown under non-irrigated conditions with tubers stored for 10 months had the highest tuber glucose content, which was greater than any other location and storage period combination. In contrast, plants grown under irrigated conditions with tubers stored for 1, 3, or 6 months had the lowest tuber glucose contents. The source of the interaction was the seven-month storage period, which had a lower tuber glucose content when plants were grown under non-irrigated compared to irrigated conditions, while the other seven storage temperatures had decreased tuber glucose contents when plants were grown under non-irrigated compared to irrigated conditions. 

Tuber glucose content was influenced by cultivar and tuber storage temperature (Table 4). ‘Dakota Diamond’ tubers stored at 7.2 °C had the highest tuber glucose content, while ‘Dakota Crisp’, ‘Dakota Diamond’ and ‘Dakota Pearl’ tubers stored at 8.9 °C had the lowest tuber glucose contents. The source of the interaction was ‘NorValley’ and ‘Dakota Diamond’ tubers stored at 7.2 and 8.9 °C. ‘Dakota Diamond’ tubers stored at 7.2 °C had higher glucose content than ‘NorValley’ tubers stored at the same temperature, while ‘Dakota Diamond’ tubers stored at 8.9 °C had lower glucose content than ‘NorValley’ tubers stored at the same temperature.

Tuber glucose content was influenced by storage temperature and storage period (Table 5). Similar to the results for sucrose, tubers stored at 7.2 °C for 10 months had the highest tuber glucose content, while tubers stored at 8.9 °C for 1–10 months had the lowest tuber glucose content. The source of the interaction was the tuber storage at 5.5 °C, where the tuber glucose content was the highest when tubers were stored for 3 months and then decreased after storage for 4 months, while tubers stored at 7.2 °C had lower tuber glucose content when stored for 3 months and increased after storage for 4 months. 

Lastly, tuber glucose content was influenced by cultivar and storage period (Table 6). “NorValley’ tubers stored for 10 months had the highest tuber glucose content, higher than the tuber glucose content for any other cultivar and storage-period combination. In contrast, ‘Dakota Pearl’ tubers stored for 1, 2, 3, 4, or 5 months had the lowest tuber glucose content. The source of the interaction was primarily ‘NorValley’ and ‘Dakota Diamond’, where ‘NorValley’ tuber glucose content increased 129% from 1.10 to 2.52 mg g^−1^ when tubers were stored for 7 and 10 months, respectively, while ‘Dakota Diamond’ tuber glucose content decreased 4% from 1.61 to 1.55 mg g^−1^ when tubers were stored for 7 and 10 months, respectively. 

### 2.3. Visual Chip Score

Chip color was measured visually using 1 (light) to 10 (dark), according to the Color Reference Standard for potato chips (courtesy of B.L. Thomas, B.L. Thomas & Associates, Cincinnati, OH, and Potato Chip Institute International). The four-way interaction of location by cultivar by storage temperature by storage period was not significant (*p* = 0.14); however, three three-way interactions and four two-way interactions were significant. 

Chip visual grade was influenced by location and storage period (Table 3). Tubers from the non-irrigated location stored for 10 months had the highest chip color score, while tubers from the irrigated location stored for 6 months had the lowest chip color score. This low chip color score was the only time that chip visual grade was acceptable, as a visual score of 4 or less is considered acceptable by the chip industry. The interaction occurred because the chip color score was generally higher for tubers grown in non-irrigated conditions compared to irrigated conditions, except when tubers were stored for 4 and 7 months. For these two storage periods, chip color scores were higher from tubers grown under irrigated conditions compared to non-irrigated.

Chip visual grade was influenced by cultivar and storage temperature (Table 4). Chips from ‘Dakota Diamond’ tubers stored at 7.2 °C had the highest chip color score, followed by ‘Dakota Diamond’ chip from tubers stored at 5.5 °C; both were the only treatments with a chip visual grade of 6.0 or higher. Chips from ‘Dakota Crisp’ tubers stored at 8.9 °C had the lowest chip color scores, and were the only chips considered acceptable by the chip industry [29]. The interaction resulted because the ‘Dakota Pearl’ chip color score was lower than the ‘Dakota Crisp’ chip color score when tubers were stored at 7.2 °C. However, at 8.9 °C the ‘Dakota Crisp’ chip color score was lower than the ‘Dakota Pearl’ chip color score.

Chip visual grade was influenced by storage temperature and storage time (Table 5). The highest chip color score occurred when tubers were stored at 7.2 °C for either 4 months or 10 months. These storage temperature and storage-period combinations were the only two combinations that resulted in visual chip color scores > 6.0. The lowest chip color scores occurred when tubers were stored at 8.9 °C for 2, 5, 6, or 7 months and were the only storage temperature and storage-period combinations that resulted in acceptable chip color by the chip industry. The interaction occurred because the chip color scores for tubers stored at 5.5 °C went from 5.6 for 1 month of tuber storage to 4.9 at 2 months of tuber storage and then back to 5.7 at three months of tuber storage, while chip color was primarily constant, at approximately 5.3 for 1, 2, and 3 months when tubers were stored at 7.2 °C.

### 2.4. Agtron Values

The four-way interaction of location by cultivar by storage temperature by storage period was not significant (*p* = 0.28). However, two significant three-way interactions of cultivar by storage temperature by storage period (*p* = 0.01) and location by storage temperature by time (*p* < 0.0001) resulted in six significant two-way interactions.

Chip Agtron values were influenced by location and cultivar (Table 1). The highest Agtron value, which was significantly greater than all others, occurred with irrigated ‘Dakota Pearl’. However, this chip color was only acceptable. The lowest Agtron value, which was significantly less than all others, occurred with non-irrigated ‘Dakota Diamond’. This was the only treatment that would have been rejected due to chip color. The interaction occurred because the ‘Dakota Crisp’ chip Agtron values slightly increased from 52.6 to 55.0 when comparing non-irrigated to irrigated production, while the ‘NorValley’ chip Agtron values had a larger increase from 51.9 to 56.4 when comparing non-irrigated to irrigated production.

Chip Agtron values were influenced by location and storage temperature (Table 2). The highest Agtron value, which was significantly greater than all others, occurred when potatoes were irrigated, and tubers were stored at 8.9 °C. In contrast, the lowest Agtron value, which was significantly less than all others, occurred when potatoes were not irrigated, and tubers were stored at 7.2 °C. This was the only combination where chips would be rejected due to their dark color. The interaction occurred because of the greater increase in Agtron values when comparing non-irrigated potatoes to irrigated potatoes with tubers stored at 5.5 °C and non-irrigated potatoes to irrigated potatoes with tubers stored at 7.2 °C.

Chip Agtron values were influenced by location and storage time (Table 3). The highest Agtron values, which were significantly greater than all others, occurred when potatoes were irrigated, and tubers were stored for only 1 or 3 months. However, irrigated potatoes stored for 1, 2, 3, 5, and 6 months had acceptable chip color. The lowest Agtron value, which was significantly less than all others, occurred when potatoes were not irrigated, and tubers were stored for 10 months; the chip color from these tubers was considered unacceptable. Similarly, when potatoes were not irrigated, and tubers were stored for 6 months, the chip color was also unacceptable. The interaction occurred because non-irrigated potatoes had an increase in chip color for tubers stored for 7 months with a subsequent decrease when examined at 10 months, while irrigated potatoes had a decrease in chip color for tubers stored for 7 months and an increase in chip color when examined at 10 months.

Chip Agtron values were influenced by cultivar and storage temperature (Table 4). The highest Agtron values were for ‘Dakota Crisp’, ‘Dakota Diamond’, and ‘Dakota Pearl’ tubers stored at 8.9 °C. All three combinations had acceptable chip color, as did ‘Dakota Pearl’ tubers stored at 5.5 °C and ‘NorValley’ tubers stored at either 5.5 °C or 8.9 °C. The lowest Agtron values occurred from ‘Dakota Diamond’ tubers stored at 5.5 °C and 7.2 °C, which were significantly less than all others. These two combinations also had the only unacceptable chip color. The interaction occurred because ‘NorValley’ chips had a greater decrease in chip color than ‘Dakota Crisp’ chips when tubers were stored at 7.2 °C and because ‘Dakota Diamond’ had a greater increase in chip color than ‘NorValley’ when tubers were stored at 8.9 °C.

Chip Agtron value was influenced by storage temperature and storage time (Table 5). The highest Agtron values, which were significantly greater than all others, occurred when tubers were stored at 8.9 °C and for 1, 2, 3, or 5 months. In contrast, the lowest Agtron values, which were significantly less than all others and considered unacceptable with respect to chip color, occurred when tubers were stored at 7.2 °C and for 7 or 10 months. Even though tubers stored at 7.2 °C and for 4 or 6 months had slightly better chip color values, these chips were also considered unacceptable. The interaction occurred because the chip color values for tubers stored at 7.2 °C were higher for the first three months compared to tubers stored at 5.5 °C and for the same storage period. However, at 4 months of storage, the chip color values for tubers stored at 7.2 °C decreased to 49.4, while the chip color values for tubers stored at 5.5 °C remained mainly stable, at 55.5.

Lastly, chip Agtron values varied between cultivar and storage period (Table 6). The highest Agtron values, which were significantly greater than all others and considered excellent with respect to chip color, occurred when ‘Dakota Pearl’ tubers were stored for 1, 2, or 3 months. In contrast, the lowest Agtron value, which was significantly less than all others, and considered unacceptable in chip color, occurred when ‘NorValley’ tubers were stored for 10 months. Even though ‘Dakota Pearl’ tubers stored for 10 months and ‘Dakota Diamond’ tubers stored for 6, 7, or 10 months had slightly better chip color values, these chips were also considered unacceptable. The interaction occurred because the chip color values for ‘NorValley tubers stored decreased from a reading of 51.1 at 7 months to 45.7 at 10 months, while the chip color values for ‘Dakota Diamond’ tubers increased from a reading of 48.2 at 7 months’ storage to 49.3 at 10 months’ storage.

## 3. Discussion

While atmospheric conditions, including CO_2_ level and ethylene, were not measured during the storage portions of this study, in retrospect these would be important components in potato storage. The USDA-ARS Potato Worksite did not have such capabilities at the time of this study, but it has since undergone a significant update. Conventional wisdom as reported in Daniels-Lake et al. [29] indicated CO_2_ accumulation in storage resulted in darker potato-fry color; however, this same work reported conflicting results in their own and previous literature. Results for short-term storage (3 and 9 w), with elevated CO_2_, reduced O_2_, and ethylene exposure, and combinations thereof, found little or no effect on fry color or tuber sugar levels for CO_2_ with or without changes in 0_2_ levels, compared to untreated controls. Ethylene alone increased tuber reducing-sugar levels, resulting in darker fry color in three of the four studies, while elevated CO_2_ in combination with ethylene resulted in darker fry color and higher reducing-sugar levels than for ethylene alone. They proposed that a synergistic negative effect may be the explanation for the often-contradictory reports in the literature. Further work by Daniels-Lake and Prange [30] found a dose–response not reported by previous authors resulting in elevated sugar levels and the darker fry colors from storage. Both studies did not examine the effects of the gases on treatments initiated at harvest, but instead were initiated after several months of storage. Daniels-Lake [31] examined long-term exposure to elevated CO_2_ and trace concentration of ethylene for 8 months’ storage (November through June) for three French fry processing cultivars and the chipping cultivar ‘Dakota Pearl’ and reported that chip color and sugar levels were minimally influenced by elevated CO_2_ and ethylene for ‘Dakota Pearl’.

### 3.1. Tuber Sucrose Content

More than 50% of potatoes produced in the US and about 65% of potatoes produced in Minnesota and North Dakota are processed, primarily into chips and French fries. High starch and low sugar content are desired, and generally result in products with acceptable texture and color attributes. Tuber reducing sugars (glucose and fructose) combine with amino acids such as asparagine in the non-enzymatic ‘Maillard’ reaction in the presence of heat. Sucrose content contributes little, initially, to color development if environmental conditions are appropriate; however, sucrose breaks into the reducing sugars due to stress resulting from environmental stress during crop growth and storage [32,33]. Tuber sucrose level was influenced by location, cultivar, storage temperature and storage period. All combinations resulted in sucrose levels exceeding the level of 1.5 mg g^−1^ fresh wt. desired by the chip processing industry at harvest and the 1.0 mg g^−1^ fresh wt. concentration during storage [34], with few exceptions (Table 1, Table 2, Table 3, Table 4, Table 5 and Table 6). Stress during the growing season, including temperature and moisture, influence tuber growth, photosynthetic rates, and carbohydrate partitioning [35,36,37]. Kleinkopf [38] concluded that potato production is best under conditions of uniform and high-water availability, warm days, and cool nights. Many authors found irregularities in source–sink relationships affecting carbohydrate synthesis and starch deposition [35,39,40,41,42,43,44,45,46]. Average air temperatures at the non-irrigated location (Prosper, ND) were below the normal average for May through August in 2004, and, conversely, near normal in 2005 (Table 7). Somewhat similarly, average air temperatures at the irrigated location (Tappen, ND) were below normal in 2004, though above normal in June and July in 2005. Precipitation in 2004 at the non-irrigated site was 63% and 11% above normal in May and July, respectively, and below normal precipitation in both June (88%) and August (48%), important times for tuber initiation and bulking, respectively. In 2005, precipitation again fluctuated every other month at the non-irrigated site. However, in May and July, precipitation was 18% and 62% below normal, while in June and August precipitation was 49% and 68% above normal, respectively. Precipitation at the irrigated site in 2004 and 2005 followed similar patterns, but was supplemented with irrigation to overcome water deficits. Environmental monitoring devices were not used in the research plots to gain specifics into stress events and tuber development stage; however, sucrose content for cultivars early in storage was above the desired level of 1.5 mg g^−1^ fresh wt. considered for chemical maturity [28], indicative of physiologically immature or overly mature tubers. High sucrose going into storage would predispose tubers in storage to increasing reducing sugar content as the sucrose is broken down. Cultivars vary in their ability to accumulate starch and sugars in tubers, and sugar concentrations differ after storage [38]. At storage temperatures from 10 °C to 12.7 °C, the balance between starch and sugars remains generally constant; however, as temperatures decrease, starches are converted to sugars. Senescent sweetening results from tuber age and is not temperature-induced. Sucrose and glucose contents climbed for cultivars, storage temperature, and storage period by the 10-month storage evaluation (Table 3, Table 5 and Table 6), indicative of senescent sweetening for all cultivars. 

### 3.2. Tuber Glucose Content

Similar to tuber sucrose, a significant four-way interaction between location, cultivar, storage temperature and storage period was found for the reducing sugar, glucose. 

Glucose levels of 0.35 mg g^−1^ fresh wt. at harvest and from storage are desired for successful chip processing of potato tubers [34]. All cultivars exhibited glucose levels exceeding the desired content under both irrigated and non-irrigated conditions (Table 1), although ‘Dakota Pearl’ had the lowest concentration at both locations and ‘Dakota Diamond’, the highest, when averaged over years, storage temperature and storage period. Cultivars differ for sugar accumulation and concentration [6,47], making studies such as this imperative in determining appropriate cultivar specific management practices. Glucose concentration was lower for potatoes produced under irrigated conditions compared to non-irrigated production at all storage temperatures (Table 1 and Table 2) when averaged over years, cultivar and storage period. This was expected, given that irrigation is designed to provide adequate moisture to developing tubers throughout the growing season and will cool both the canopy and soil in periods of high temperature stress. Tubers stored at 8.9 °C had a mean glucose content within the desired range for processing (Table 2). Similarly, glucose content was lower for all storage periods (Table 3) when tubers were produced under irrigation. However, no levels met the industry threshold. ‘Dakota Pearl’ had the lowest glucose levels at each storage temperature (Table 4); it is considered a cold-chipping cultivar [47], although at 8.9 °C all cultivars had their lowest values when average over years, location, and storage period. Similarly, all storage periods had lower glucose contents for warmer storage temperatures (Table 5). Generally, tuber glucose increased with storage period for cultivars (Table 6), although no combinations met the industry threshold. When storage temperatures are too low for a cultivar, both sucrose and glucose levels will increase [34]. Barichello et al. [48] found an increased respiration rate for clones resistant to temperature sweetening. Our study did not measure respiration, nor the enzymes associated with low-temperature sweetening; however, respiration tends to be higher as storage temperature increases across genotypes, resulting in respiration of some of the sugar components as energy [1]. 

### 3.3. Visual Chip Color

Unlike the sucrose and glucose contents, the four-way interaction between location, cultivar, storage temperature, and storage period were not significant; however, there were significant three-way and two-way interactions (Table 1, Table 2, Table 3, Table 4, Table 5 and Table 6). Visual chip color is related to the glucose content, particularly due to the interaction of heat, reducing sugars, and amino acids in the Maillard reaction [49]. Sucrose plays a lesser role in chip color. Utilizing the Color Reference Standard for Potato Chips (courtesy of B.L. Thomas, B.L. Thomas & Associates, Cincinnati, OH, and the Potato Chip Institute International), a visual assessment score of 4 or less is considered acceptable for chip processing. Chip color was not acceptable for chips of any cultivars when grown under irrigated or non-irrigated conditions (Table 1). Chips from all locations for storage period were only acceptable from tubers produced under irrigation and stored for 6 months (Table 3). The most unacceptable chips resulted from tubers produced under non-irrigated conditions and storage for 10 months. Across factors, only ‘Dakota Pearl’ chips from the 5- and 6-month storage periods were acceptable (Table 6), although ‘Dakota Crips’ chips were acceptable from the warmest storage temperature of 8.9 °C (Table 4). The warmest storage temperature of 8.9 °C produced the lightest-colored chips for all storage periods (Table 5). Discussion of the sugar-component results above indicate elevated sucrose and glucose levels throughout storage, with sugar components increasing with time. Increasing sugars, particularly glucose, results in darker-colored fried products, and a corresponding increase in visual chip scores.

### 3.4. Agtron Value

Agtron values are an unbiased assessment of chip color. Similar results for the visual chip color assessment were found, with several two- and three-way interactions significant, although the four-way interaction of location, cultivar, storage temperature, and storage period was not significant. Agtron values of 60 or higher (scale 1–100, dark to light) are desired by the chip processing industry. Agtron values higher than 60 were not attained for most factors (Table 1, Table 2, Table 3, Table 4, Table 5 and Table 6). Ratings of 56 to 60 are considered acceptable, while values of 50 to 55 are considered marginal. ‘Dakota Diamond’ tubers produced under non-irrigated conditions were unacceptable (Table 1). ‘Dakota Diamond’ is a late-maturing cultivar, often over-fertilized, and thus harvested when chemically immature [19]. High glucose concentrations would result in unacceptably dark chips. The visual scores also reflected this, as per the discussion above. Agtron reflectance values were also unacceptable for chips processed from tubers produced under non-irrigated from the 7.2 °C storage temperature (Table 2); however, chips were marginal-to-acceptable for other locations by storage temperature combinations. Chips processed from tubers produced under irrigated conditions were lighter than those from tubers produced under non-irrigated conditions, while shorter storage resulted in lighter chips and higher Agtron values (Table 3) for both locations. As storage time increased, sugars increased, leading to darker chip colors and, thus, lower Agtron values. ‘Dakota Pearl’ from all temperatures except for chips of ‘Dakota Crisp’ from 8.9 °C storage was lightest and had correspondingly higher Agtron values (Table 4). These results would confirm the cold-chipping ability of ‘Dakota Pearl’ [48]. Interestingly, Agtron reflectance values were lowest from 7.2 °C storage for storage periods 4 through 10 months (Table 5), and highest for chips from 8.9 °C storage through the 6-month storage period. Generally, colder storage temperatures resulted in higher sugar content over time, and correspondingly darker chip scores, resulting in lower Agtron reflectance values, while warmer storage temperatures resulted in less starch-to-sugar interconversion and breakdown of sucrose to its reducing sugar components, glucose and fructose. However, sucrose levels were high initially, and, across storage temperatures and storage period, tended to increase in our study. The initially high sucrose levels may be due to environmental stress, including high temperature and low moisture availability, or may be due to chemical immaturity due to nitrogen availability and/or vine maturity. Vine maturity was not rated, but may have helped explain differences in sugar components and subsequent visual chip color and reflectance scores. As storage period increased, all cultivars had chip samples with lower Agtron values (Table 6). ‘Dakota Pearl’ had the highest Agtron values across the early storage periods, and had the lowest glucose contents of all cultivars at those storage periods, as well. Correlation between visual chip scores and Agtron reflectance values was not conducted; however, it would have been interesting to learn if visual assessment is adequate, given the speed at which it can be obtained, versus the added step of an unbiased instrument. Few chipping labs utilize Agtron values today, in favor of newer colorimeters which provide lightness and chromaticity (brightness, for example) attributes.

## 4. Materials and Methods

### 4.1. Field Experiment Description

Research plots were planted at the Northern Plains Potato Growers Irrigated Research site near Tappen, ND, USA on an Arvilla sandy loam soil (sandy, mixed, frigid Calcic Hapludoll) under irrigation, and at the North Dakota State University research site near Prosper, ND, USA with a Perella–Bearden silty clay loam soil (Perella: fine-silty, mixed, superactive Typic Endoaquoll; Bearden: fine-silty, mixed superactive, frigid Aeric Calciaquoll) for the non-irrigated study, during 2004 and 2005. Field experiments were arranged as a randomized complete block with four replicates. Treatments included a factorial arrangement of three nitrogen rates (112, 168 and 224 Kg ha^−1^) and four potato cultivars (Dakota Crisp, Dakota Diamond, Dakota Pearl, and NorValley) at both sites [50]. The middle two rows of each plot were harvested approximately 21 days after the first vine desiccant application of diquat dibromide 1.06 kg ai ha^−1^ (Reglone^®^ 2.37 L ha^−1^). Total yield, tuber size, and tuber numbers per plot were determined using a programmable electronic sizer located at the USDA-ARS Potato Worksite at East Grand Forks, MN, USA. Tubers were graded into > or <113 g, to determine the marketable yield. Tubers less than 113 g are undesirable for chipping because of their small size [51,52].

### 4.2. Storage Experimental Description

Storage experiments were arranged as a four-factor completely randomized design with cultivar (Dakota Crisp, Dakota Diamond, Dakota Pearl, NorValley) and nitrogen rate (112, 168, 224 kg ha^−1^) as one factor. Nitrogen rate was not included as a factor because nitrogen rate did not influence chip color at harvest [50]. Location (irrigated or non-irrigated) was the second factor, storage period (1, 2, 3, 4, 5, 6, 7 and 10 months) was the third factor, and storage temperature (5.5, 7.2, 8.9 °C) was the fourth factor. 

Each year, eight good-quality marketable tubers (each tuber weight > 113 g) were selected after grading (avoiding rotten, green, or damaged tubers) from both sites (irrigated and non-irrigated) and placed into a mesh bag for storage. Thirty samples were saved from each plot to ensure sufficient sample numbers for three storage temperatures and the ten-month storage period. Potato samples were preconditioned at approximately 13 °C for 1 month following harvest to allow for wound healing, prior to decreasing the temperature in treatment coolers to approximately 5.5 °C (4.9–6.1 °C), 7.2 °C (6.7–7.8 °C), or 8.9 °C (8.3–9.4 °C) and maintaining 95% relative humidity in each cooler. Each cooler was 3.4 m by 2.4 m by 3.0 m (LxWxH). Samples were assessed monthly in 2004, but were processed at bimonthly intervals in 2005, to determine sugar components and chip color from storage.

### 4.3. Color Measurement

Tubers from each sample were cut in half lengthwise (bud to stem end); one-half of each tuber was used for sugar extraction and the remaining half for processing into chips. Only the second and third full slice from the center of each tuber was used to make up the sixteen chips for visual color rating and Agtron measurements; chips were cut to a thickness of 1.3 mm. All tuber slices were fried in vegetable oil at 185 °C for 90 s. Chip color was measured visually using 1 (light) to 10 (dark) according to the Color Reference Standard for potato chips (courtesy of B.L. Thomas, B.L. Thomas & Associates, Cincinnati, OH, USA and the former Potato Chip Institute International, Washington, DC, USA). Reference reflectance disks were used to calibrate the Agtron before measurement (Agtron value 0 = black and Agtron value 90 = white). Each sample was crushed and placed in a cuvette for Agtron value determination with an Agtron process analyzer that utilizes reflectance spectrophotometry. A score of 4 or less for visual color rating was considered an acceptable chip color value by industry standards [16]. A reading of 50 or greater for the Agtron value was considered an acceptable chip color [52]. The Agtron value for chip color was divided into more detailed categories: excellent > 60, acceptable = 56 to 60, marginal = 50 to 55, and rejected < 50 [53].

### 4.4. Sugar Extraction and Analysis

A procedure developed by researchers at the USDA-ARS Potato Storage Research site at East Grand Forks, MN, was used to extract potato tuber solutions for sugar analysis [32]. In this procedure, one-half of each tuber per sample was cored shortly after removing it from the cooler to obtain an approximately 25 g piece from each tuber until a total sample weight of 200 g (±0.2 g) was obtained. The 200 g sample was added to a juicerator (Model #6001, Waring, East Windsor, NJ, USA) with 150 mL diluted phosphate buffer, PH 7.2. (pH value was adjusted with solid NaOH, 1:1 diluted buffer with super-Q water, and stored in a cooler at 4 °C near the juicerator.) After solution extraction, diluted phosphate buffer was added to bring the final volume to 275 mL. 

Measurements of sucrose and glucose were made with the YSI Model 2700 Industrial Sugar Analyzer (Yellow Spring Instrument Co., Inc., Yellow-Springs, OH, USA). Three consecutive values were taken and averaged to determine the concentration of sucrose and glucose for each sample.

### 4.5. Statistical Analyses

Before datasets were subjected to the analysis of variance, the PROC UNIVARIATE in SAS (Version 9.4, SAS Analytics Software, Cary, NC, USA) was fitted with probability density curves and with kernel density estimates to check normal test normality, and PROC GLIMMIX in SAS was executed for a specific distribution in Model OPTIONs LINK for all datasets; the ILINK for LS means test at (alpha = 0.05) was used for means separation main and interaction effects of fixed-effect factors. Data for location were a combination of location and year, while data for cultivar were a combination of cultivar and nitrogen rate, as neither year nor nitrogen rate influenced the variables analyzed. All data were analyzed using the Glimmix procedure in SAS (Version 9.4, SAS Analytics Software, Cary, NC, USA) to test normality, make necessary transformations, and evaluate main and interaction effects. The LS means test at *p* = 0.05 was used for means separation, when appropriate.

## 5. Conclusions

Significant interactions were found for location (irrigated and non-irrigated potato production), storage temperature, storage period and cultivar affecting tuber sucrose and glucose levels, and the resulting chip processing quality assessments. ‘Dakota Crisp’, ‘Dakota Diamond’ and ‘NorValley’ all produced higher sugar levels and resulted in darker chip colors when stored at colder temperatures than the chip industry standard cultivar ‘Dakota Pearl’. ‘Dakota Crisp’ demonstrated potential for producing acceptable chip color after long-term storage at 8.9 °C, while ‘Dakota Diamond’ demonstrated the least potential for acceptable chips from long-term storage when grown under irrigated or non-irrigated conditions. Storage management strategies must minimize sugar accumulation, particularly the reducing sugar glucose, in order to maximize chip quality color attributes including visual color ratings and chip reflectance values.

## Figures and Tables

**Table 1:** Influence of location and cultivar on tuber sucrose content, tuber glucose content, chip visual color, and Agtron value averaged over storage temperature, storage time, and years.

Location	Cultivar	Sucrose Content	Glucose Content	Chip Visual Color	Agtron Value
		(mg g^−1^)	(mg g^−1^)	(1–10) ^1^	(0–100) ^2^
Irrigated	Dakota Crisp	2.65 a ^3^	0.72 c	4.7	55.0 c
Irrigated	Dakota Diamond	1.76 c	1.26 b	5.4	52.3 d
Irrigated	Dakota Pearl	2.09 b	0.45 d	4.4	57.4 a
Irrigated	NorValley	1.90 c	0.77 c	4.6	56.4 b
Non-irrigated	Dakota Crisp	1.75 c	1.22 b	5.0	52.6 d
Non-irrigated	Dakota Diamond	1.56 d	1.80 a	5.6	49.9 e
Non-irrigated	Dakota Pearl	2.21 b	0.83 c	4.7	55.4 c
Non-irrigated	NorValley	1.47 d	1.79 a	5.0	51.9 d
*p*-Value		<0.0001	<0.0001	0.0631	<0.0001

^1^ Color Reference Standard for Potato Chips (courtesy of B.L. Thomas, B.L. Thomas & Associates, Cincinnati, OH, and Potato Chip Institute International): 1–10 rating, with 1 = light and 10 dark. A visual score of 4 or less is considered acceptable by the chip industry. ^2^ Agtron value 0 = black and Agtron value 90 = white. An Agtron reading of 50 or greater was considered acceptable (excellent > 60, acceptable = 56 to 60, marginal 50 to 55, and rejected < 50). ^3^ Means with the same letter within a column are not significantly different using LS means at *p* ≤ 0.05.

**Table 2:** Influence of location and storage temperature on tuber sucrose content, tuber glucose content, chip visual color, and Agtron values averaged over cultivars, storage time and years.

Location	Storage Temperature	Sucrose Content	Glucose Content	Chip Visual Color	Agtron Value
	(°C)	(mg g^−1^)	(mg g^−1^)	(1–10) ^1^	(0–100) ^2^
Irrigated	5.5	2.10 c ^3^	0.84	4.9	55.3 c
Irrigated	7.2	3.02 a	1.23	5.4	51.8 d
Irrigated	8.9	1.17 d	0.32	4.1	58.7 a
Non-irrigated	5.5	1.52 d	1.58	5.2	51.3 d
Non-irrigated	7.2	2.24 b	1.96	5.7	49.7 e
Non-irrigated	8.9	1.50 d	0.72	4.3	56.3 b
*p*-Value		<0.0001	0.5032	0.8291	0.0003

^1^ Color Reference Standard for Potato Chips (courtesy of B.L. Thomas, B.L. Thomas & Associates, Cincinnati, OH, and Potato Chip Institute International): 1–10 rating with 1 = light and 10 dark. A visual score of 4 or less is considered acceptable by the chip industry. ^2^ Agtron value 0 = black and Agtron value 90 = white. An Agtron reading of 50 or greater was considered acceptable (excellent > 60, acceptable = 56 to 60, marginal 50 to 55, and rejected < 50). ^3^ Means with the same letter within a column are not significantly different using LS mMeans at *p* ≤ 0.05.

**Table 3:** Influence of location and storage time on tuber sucrose content, tuber glucose content, chip visual color, and Agtron values averaged over locations, storage temperature and years.

Location	Storage Period	Sucrose Content	Glucose Content	Chip Visual Color	Agtron Value
	(month)	(mg g^−1^)	(mg g^−1^)	(1–10) ^1^	(0–100) ^2^
Irrigated	1	1.95 d ^3^	0.48 i	4.7 fg	59.4 a
Irrigated	2	1.75 e	0.71 gh	4.5 gh	57.6 b
Irrigated	3	2.43 b	0.65 hi	4.5 gh	60.0 a
Irrigated	4	2.14 c	0.92 fg	5.5 c	55.2 de
Irrigated	5	1.86 de	0.70 gh	4.5 gh	56.9 bc
Irrigated	6	1.86 de	0.70 g-i	3.9 i	51.2 h
Irrigated	7	2.39 b	1.21 c–e	5.2 d	48.7 i
Irrigated	10	2.42 b	1.04 ef	5.4 cd	53.4 fg
Non-irrigated	1	1.46 f	1.15 de	5.5 c	54.3 ef
Non-irrigated	2	1.57 f	1.35 b–d	4.8 ef	55.8 d
Non-irrigated	3	1.46 f	1.55 b	5.8 b	56.1 cd
Non-irrigated	4	1.57 f	1.25 c–e	4.8 e	53.6 f
Non-irrigated	5	1.47 f	1.47 bc	4.6 fg	53.5 fg
Non-irrigated	6	1.42 f	1.26 c–e	4.4 gh	49.9 i
Non-irrigated	7	1.37 f	0.99 ef	4.3 h	52.2 gh
Non-irrigated	10	3.66 a	2.33 a	6.5 a	44.1 j
*p*-Value		<0.0001	<0.0001	<0.0001	<0.0001

^1^ Color Reference Standard for Potato Chips (courtesy of B.L. Thomas, B.L. Thomas & Associates, Cincinnati, OH, and Potato Chip Institute International): 1–10 rating with 1 = light and 10 dark. A visual score of 4 or less is considered acceptable by the chip industry. ^2^ Agtron value 0 = black and Agtron value 90 = white. An Agtron reading of 50 or greater was considered acceptable (excellent > 60, acceptable = 56 to 60, marginal 50 to 55, and rejected < 50). ^3^ Means with the same letter within a column are not significantly different using LS means at *p* ≤ 0.05.

**Table 4:** Influence of cultivar and storage temperature on tuber sucrose content, tuber glucose content, chip visual color, and Agtron values averaged over locations, and storage period, and years.

Cultivar	Storage Temperature	Sucrose Content	Glucose Content	Chip Visual Color	Agtron Value
	(°C)	(mg g^−1^)	(mg g^−1^)	(1–10) ^1^	(0–100) ^2^
Dakota Crisp	5.5	2.25 d ^3^	1.10 de	5.2 d	52.2 f
Dakota Crisp	7.2	3.11 a	1.47 c	5.5 c	51.0 g
Dakota Crisp	8.9	1.24 gh	0.39 i	4.0 i	58.2 a
Dakota Diamond	5.5	1.72 f	1.92 b	6.0 b	48.3 h
Dakota Diamond	7.2	2.07 de	2.15 a	6.3 a	47.3 h
Dakota Diamond	8.9	1.18 h	0.51 hi	4.3 gh	57.8 ab
Dakota Pearl	5.5	2.06 e	0.64 gh	4.4 fg	56.9 bc
Dakota Pearl	7.2	2.84 b	0.92 ef	5.0 e	54.1 e
Dakota Pearl	8.9	1.45 f	0.36 i	4.2 h	58.0 a
NorValley	5.5	1.21 gh	1.18 d	4.6 f	55.8 d
NorValley	7.2	2.48 c	1.84 b	5.5 c	50.6 g
NorValley	8.9	1.36 g	0.82 fg	4.3 gh	56.1 cd
*p*-Value		<0.0001	<0.0001	<0.0001	<0.0001

^1^ Color Reference Standard for Potato Chips (courtesy of B.L. Thomas, B.L. Thomas & Associates, Cincinnati, OH, and Potato Chip Institute International): 1–10 rating with 1 = light and 10 dark. A visual score of 4 or less is considered acceptable by the chip industry. ^2^ Agtron value 0 = black and Agtron value 90 = white. An Agtron reading of 50 or greater was considered acceptable (excellent > 60, acceptable = 56 to 60, marginal 50 to 55, and rejected < 50). ^3^ Means with the same letter within a column are not significantly different using LS means at *p* ≤ 0.05.

**Table 5:** Influence of storage temperature and storage time on tuber sucrose content, tuber glucose content, chip visual color, and Agtron values averaged over cultivars, locations and years.

Storage Temperature	Storage Period	Sucrose Content	Glucose Content	Chip Visual Color	Agtron Value
(°C)	(month)	(mg g^−1^)	(mg g^−1^)	(1–10) ^1^	(0–100) ^2^
5.5	1	2.56 c ^3^	1.17 fg	5.6 b	53.6 f
5.5	2	2.11 ef	1.28 e–g	4.9 d	54.4 d–f
5.5	3	1.64 hi	1.56 cd	5.7 b	55.3 cd
5.5	4	1.77 gh	1.11 f–h	4.9 d	55.5 cd
5.5	5	1.61 hi	1.14 f–h	4.7 de	54.6 d–f
5.5	6	1.50 ij	1.11 f–h	4.2 f	50.4 gh
5.5	7	1.49 i–k	1.01 gh	4.7 de	51.7 g
5.5	10	1.80 gh	1.29 d–g	5.8 b	50.9 g
7.2	1	1.52 i	0.92 h	5.3 c	56.3 c
7.2	2	1.94 fg	1.36 d–f	5.2 c	55.1 de
7.2	3	3.03 b	1.29 d–g	5.3 c	57.9 b
7.2	4	2.55 c	1.66 bc	6.3 a	49.4 h
7.2	5	2.20 de	1.71 bc	5.4 c	50.7 gh
7.2	6	2.42 cd	1.49 c–e	4.8 de	47.6 i
7.2	7	2.96 b	1.90 b	5.9 b	44.9 j
7.2	10	4.40 a	2.44 a	6.4 a	44.1 j
8.9	1	1.04 lm	0.34 i	4.3 f	60.6 a
8.9	2	0.93 m	0.45 i	3.9 g	60.7 a
8.9	3	1.16 l	0.44 i	4.6 e	60.7 a
8.9	4	1.25 j–l	0.47 i	4.3 f	58.2 b
8.9	5	1.19 l	0.41 i	3.5 h	60.2 a
8.9	6	1.01 lm	0.34 i	3.5 h	53.8 ef
8.9	7	1.20 kl	0.39 i	3.7 gh	54.7 d–f
8.9	10	2.91 b	1.32 d–g	5.7 b	51.3 g
*p*-Value		<0.0001	<0.0001	<0.0001	<0.0001

^1^ Color Reference Standard for Potato Chips (courtesy of B.L. Thomas, B.L. Thomas & Associates, Cincinnati, OH, and Potato Chip Institute International): 1–10 rating with 1 = light and 10 dark. A visual score of 4 or less is considered acceptable by the chip industry. ^2^ Agtron value 0 = black and Agtron value 90 = white. An Agtron reading of 50 or greater was considered acceptable (excellent > 60, acceptable = 56 to 60, marginal 50 to 55, and rejected < 50). ^3^ Means with the same letter within a column are not significantly different using LS means at *p* ≤ 0.05.

**Table 6:** Influence of cultivar and storage time on tuber sucrose content, tuber glucose content, chip visual color, and Agtron values averaged over locations, storage temperature and years.

Cultivar	Storage Period	Sucrose Content	Glucose Content	Chip Visual Color	Agtron Value
	(month)	(mg g^−1^)	(mg g^−1^)	(1–10) ^1^	(0–100) ^2^
Dakota Crisp	1	1.92 e–g ^3^	0.77 i–l	5.5 d–f	55.0 de
Dakota Crisp	2	1.97 d–f	0.96 g–i	4.8 j–m	55.6 d
Dakota Crisp	3	2.10 c–e	0.87 h–k	5.0 h–j	58.1 bc
Dakota Crisp	4	2.22 cd	1.05 g–i	5.2 f–h	54.1 ef
Dakota Crisp	5	2.00 d–f	1.02 g–i	4.4 o–r	54.8 d–f
Dakota Crisp	6	1.94 d–g	0.86 h–k	4.1 r–t	50.8 h–j
Dakota Crisp	7	2.41 c	0.96 g–j	4.6 m–q	51.3 hi
Dakota Crisp	10	3.04 b	1.40 b–f	5.5 d–f	50.6 h–k
Dakota Diamond	1	1.71 g–i	1.31 d–g	5.7 c–e	53.5 fg
Dakota Diamond	2	1.60 h–j	1.63 bc	5.4 fg	52.1 gh
Dakota Diamond	3	1.81 f–h	1.59 b–d	6.0 bc	54.0 ef
Dakota Diamond	4	1.55 ij	1.65 b	5.8 c–e	51.1 h–j
Dakota Diamond	5	1.47 i–k	1.45 b–e	5.2 g–i	52.0 h
Dakota Diamond	6	1.40 j–l	1.42 b–f	4.7 l–o	48.8 kl
Dakota Diamond	7	1.63 g–j	1.61 b–d	5.3 f–h	48.2 l
Dakota Diamond	10	2.08 c–f	1.55 b–d	6.1 ab	49.3 j–l
Dakota Pearl	1	1.99 d–f	0.34 m	4.3 p–r	60.7 a
Dakota Pearl	2	1.83 f–h	0.62 j–m	4.1 r–t	60.8 a
Dakota Pearl	3	2.16 cd	0.56 lm	4.9 i–l	60.7 a
Dakota Pearl	4	2.19 cd	0.55 lm	4.6 l–p	57.5 d
Dakota Pearl	5	1.68 g–j	0.57 k–m	4.0 st	58.7 bc
Dakota Pearl	6	1.64 g–i	0.52 lm	3.8 t	52.0 gh
Dakota Pearl	7	2.02 d–f	0.73 i–l	4.6 l–p	51.1 h–j
Dakota Pearl	10	3.70 a	1.26 d–h	5.9 bc	49.5 i–l
NorValley	1	1.20 l	0.82 i–l	4.8 k–n	58.2 bc
NorValley	2	1.23 kl	0.91 h–j	4.3 q–s	58.4 bc
NorValley	3	1.69 g–j	1.37 b–f	4.9 i–m	59.2 b
NorValley	4	1.46 i–l	1.08 f–i	5.0 h–k	54.9 de
NorValley	5	1.47 ij	1.31 c–g	4.5 n–q	55.3 de
NorValley	6	1.59 h–j	1.12 e–i	4.1 r–t	50.7 h–j
NorValley	7	1.47 i–l	1.10 e–i	4.6 l–p	51.1 h–j
NorValley	10	3.34 b	2.52 a	6.3 a	45.7 m
*p*-Value		<0.0001	<0.0001	<0.0001	<0.01

^1^ Color Reference Standard for Potato Chips (courtesy of B.L. Thomas, B.L. Thomas & Associates, Cincinnati, OH, and Potato Chip Institute International): 1–10 rating with 1 = light and 10 dark. A visual score of 4 or less is considered acceptable by the chip industry. ^2^ Agtron value 0 = black and Agtron value 90 = white. An Agtron reading of 50 or greater was considered acceptable (excellent > 60, acceptable = 56 to 60, marginal 50 to 55, and rejected < 50). ^3^ Means with the same letter within a column are not significantly different using LS means at *p* ≤ 0.05.

**Table 7:** Average monthly air temperatures and total monthly precipitation at the non-irrigated (Prosper, ND, USA) and irrigated (Tappen, ND, USA) potato production locations during 2004 and 2005.

	Non-Irrigated (Prosper, ND, USA)				Irrigated (Tappen, ND, USA)			
2004	Av. Air Temp. (°C)	Normal Av. Air Temp. (°C)	Total Precip. (mm)	Normal Total Precip. (mm)	Av. Air Temp. (°C)	Normal Av. Air Temp. (°C)	Total Precip. (mm)	Normal Total Precip. (mm)
May	11	13	127.5	78.0	10	12	120.9	74.9
June	16	19	13.2	108.5	15	17	70.9	88.1
July	19	21	101.1	90.9	19	20	55.1	84.6
August	16	20	35.1	67.0	16	19	31.2	61.5
2005								
May	12	13	64.0	78.0	11	12	70.4	74.9
June	20	19	161.3	108.5	19	17	103.9	88.1
July	21	21	34.3	90.9	22	20	50.3	84.6
August	19	20	112.5	67.1	19	19	47.2	61.5

## Data Availability

Data can be made available by contacting the corresponding author.
